# Analysis of the Impact of Preadmission, Inpatient, and Discharge Opioid Exposure and Dose on 30- and 90-Day Hospital Readmissions in Patients with Inflammatory Bowel Disease Exacerbations

**DOI:** 10.3390/jcm14217658

**Published:** 2025-10-28

**Authors:** Ellen A. Oseni, Miriam Blumenthal, Stephanie Izard, Michael Qiu, Anjali Mone, Arun Swaminath, Keith Sultan

**Affiliations:** 1 Division of Gastroenterology, Northshore University Hospital, Manhasset, NY 11030, USA; mblumenthal@northwell.edu (M.B.); ksultan@northwell.edu (K.S.); 2 Quantitative Intelligence, Feinstein Institutes for Medical Research, Manhasset, NY 11030, USAmqiu@northwell.edu (M.Q.); 3 Division of Gastroenterology, Lenox Hill Hospital, New York, NY 10075, USA; amone1@northwell.edu (A.M.); aswaminath@northwell.edu (A.S.)

**Keywords:** inflammatory bowel disease, Crohn’s disease, ulcerative colitis, opioids, readmissions

## Abstract

**Background:** Opioid use is common among patients hospitalized for inflammatory bowel disease (IBD) exacerbation and has been associated with an increased risk of readmissions. Prior studies, however, have mostly limited their analysis to hospital opioid use. This study examines opioid exposure and dosing before, during, and after hospitalization and its impact on 30- and 90-day hospital readmissions. **Methods:** We reviewed all adults admitted for an IBD exacerbation from 1 January 2016 to 3 January 2020, excluding pregnant patients and those with an IBD-related surgery. Use and dose of opioids before and during hospitalization and at discharge were identified through manual chart review. IBD type, demographics, and comorbidities were defined. The associations between opioid use characteristics and readmission were assessed using a series of multivariable logistic regression models. **Results:** Among 1062 patients meeting inclusion criteria, 191 (18.0%) were readmitted within 30 days of their index hospitalization, and 285 (26.8%) were readmitted within 90 days. Of these 1062 patients, 96 (9.02%) had preadmission opioid use, 340 (31.95%) had inpatient use, and 133 (12.50%) received a discharge opioid prescription. Preadmission, inpatient, and discharge opioid use were not associated with 30-day readmission. Preadmission and inpatient opioid use were also not associated with 90-day readmission; however, a prescription for an opioid at discharge was associated with 90-day readmission even after adjusting for confounders, OR 1.86 (1.27, 2.75), *p* = 0.002. On multivariable analysis, we also found that higher maximum daily dose of discharge opioids, OR 1.01 (1.00, 1.02), *p* = 0.037, was found to be associated with 30-day readmissions, and higher opioid doses preadmission, OR 1.01 (1.00, 1.03), *p* = 0.029, and at hospital discharge, OR 1.01 (1.00, 1.02), *p* = 0.034, were associated with increased 90-day readmission. **Conclusions:** Opioid prescribing at discharge poses a significant risk for readmissions. Discharge planning should emphasize minimal use of opioids at discharge.

## 1. Introduction

Inflammatory bowel disease (IBD), mostly categorized as Crohn’s disease (CD) or ulcerative colitis (UC), is a chronic inflammatory condition that may be associated with acute flares or complications that require hospitalization and, in certain cases, hospital readmission. The underlying indications for hospitalization are diverse and include common IBD complaints such as abdominal pain, diarrhea, gastrointestinal bleeding, fever, and general failure (or lack) of outpatient medical care. The negative effects of these hospitalizations on quality of life (QOL) are well established, as well as their association with morbidity and mortality among this population [1,2]. These findings speak to the importance of understanding and addressing factors associated with hospital readmissions among IBD patients.

Despite therapeutic advancements, the management of pain among IBD patients continues to pose a significant challenge. Many studies have addressed the prevalence of opioid use and an increase in opioid prescriptions among IBD patients [3,4,5], and have even associated high-dose opioid use with an increase in all-cause premature mortality [6]. Despite its widespread use, the therapeutic benefits of opioid use in IBD-related pain remain controversial, and its use is generally discouraged. A recent study by Berry et al. emphasized this concern by examining the impact of opioids on pain among hospitalized IBD patients [7]. Findings showed inadequate pain control during hospitalization, with patients reporting insignificant changes in pain from admission to discharge despite opioid exposure.

Avoidance of short-term readmission is a key goal of any hospitalization. Among the many factors associated with IBD hospital readmissions, there is evidence to suggest that opioid use is a significant risk factor for hospitalization, longer inpatient lengths of stay, and readmissions [3,4,8,9,10,11]. While others have investigated this issue, few have evaluated the complete spectrum and relative impact of preadmission, inpatient, and discharge opioid use on hospital readmission, nor the impact of the actual opioid dose used on hospital admission or readmission. Our goal was to comprehensively examine a cohort of patients admitted for an IBD exacerbation, define opioid exposure, and calculate opioid dosing estimates before, during, and after hospitalization and their association with 30-day and 90-day hospital readmissions.

## 2. Methods

### 2.1. Study Design, Inclusion and Exclusion Criteria

IRB approval was obtained for a retrospective chart review of all adult IBD patients over the age of 18 who were admitted through the emergency room (ER) and underwent hospitalization for an IBD exacerbation from 1 January 2016 to 3 January 2020 within the Northwell Health Care system. Patients were identified by either a primary or secondary ICD-10 code (K50.xx, K51.xx), corresponding to CD or UC. The sample was limited to patients with an IBD exacerbation defined by administration of intravenous (IV) solumedrol and/or biologic therapy during the index admission. For patients with multiple admissions during the study period, the first recorded admission was regarded as the index hospitalization, limiting each patient analyzed to one index admission. Pregnant patients and those who underwent an IBD-related surgery during the index admission were excluded from analysis.

### 2.2. Variables of Interest

Patient demographic and clinical characteristics examined included age (years), gender (female, male), disease type (CD, UC), race (African American/Black, Asian, Other/Multiracial, White), tobacco use (current, former, never, unknown), and alcohol use (current, former, never, unknown), a diagnosis of anxiety or depression, Charlson Comorbidity Index (CCI) score, and index admission length of stay (LOS) in days.

Opioid use was evaluated separately as preadmission, inpatient, and discharge opiate use. Opioid dosing was estimated for all patients with opiate use, and differing opioid formulations were standardized by their morphine milligram equivalents (MME). Home opioid maximum daily dose was defined as the total maximum daily dose per day of all oral (PO) opioids per subject. Inpatient opioid average daily dose was calculated by summing all opioid dose amounts (IV, PO, and TD types) and dividing by inpatient length of stay. Modes of inpatient opioid administration (IV, PO, and TD) were estimated separately since patients could have multiple modes of opioid administration within the same visit. Discharge opioid maximum daily dose was defined as the total maximum daily dose prescribed at the time of discharge per day of all PO opiates per subject. The primary outcomes of interest were 30-day and 90-day all-cause readmission from the index admission. Ninety-day readmissions included those readmitted within 30 days of hospital discharge, along with those additional patients readmitted between days 31 and 90 post-discharge. Analysis was limited to a single readmission event following the first/index admission for each patient.

### 2.3. Statistical Analysis

All demographic and clinical characteristics were summarized descriptively. Categorical variables were summarized across readmission categories using frequency and percentage, and continuous variables were summarized across readmission categories using median and interquartile range. Descriptive statistics were performed to determine if any characteristics significantly differed by readmission category, using the chi-square test (or Fisher’s Exact test when >20% cell counts were <5) for categorical variables and the Wilcoxon Rank Sum test for continuous variables. These comparisons were also used as a bivariate screen to identify confounders to be included as covariates in our adjusted models moving forward (*p* < 0.05).

The univariable association between each exposure of interest and the 30- and 90-day readmission outcomes was first assessed using descriptive statistics similarly to the demographic and clinical characteristics of interest. A series of multivariable logistic regression models was used to determine the association between each exposure and outcome of interest, adjusting for CCI and LOS, as identified in the univariable screen detailed above. All results were considered significant at *p* < 0.05, and all analyses were performed in R Version 4.1.2.

This study was conducted in compliance with the ethical standards of Northwell Health for human subjects as well as with the Helsinki Declaration.

## 3. Results

### 3.1. Patient Characteristics

There were 1062 patients who met the inclusion criteria, with a median age of 43.0 years, interquartile range (IQR) 28.0 years to 61.0 years, of whom 501 (47.2%) had CD and 537 (50.6%) were female (Table 1).

**Figure 1:**
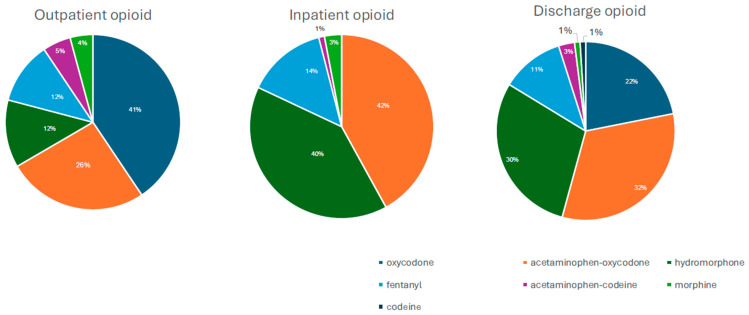
Distribution of opioid types administered in inpatient, discharge and outpatient settings.

Overall, 96 (9.0%) patients were using opioids prior to hospitalization, 340 (32.0%) received opioids during hospitalization, and 132 (12.4%) were discharged from their index hospitalization with a prescription for continued opioid use. Oxycodone alone, or in combination with acetaminophen, was the most commonly used opioid before and during hospitalization and at the time of discharge (Figure 1).

### 3.2. Opioid Use and 30-Day Readmission

There were 191 patients with a 30-day readmission. Of these patients, 25 (13.1%) had preadmission opioid use, 70 (36.6%) had inpatient opioid use, and 34 (17.8%) were discharged with an opioid prescription. On univariable analysis, 30-day readmission was found to be associated with preadmission (*p* = 0.031) and discharge opioid use (*p* = 0.013), but not with inpatient opioid use (*p* = 0.130) (Table 2).

After adjusting for CCI and LOS, preadmission, inpatient, and discharge opioid use were not found to be associated with 30-day readmission (Table 2).

### 3.3. Opioid Use and 90-Day Readmission

There were 285 patients with a 90-day readmission. Of these, 34 (11.9%) had preadmission opioid use, 100 (35.1%) had inpatient opioid use, and 54 (18.9%) had discharge opioid use. In unadjusted analyses, 90-day readmission was found to be associated with preadmission (*p* = 0.047) and discharge (*p* < 0.001) opioid use, but not with inpatient opioid use (*p* = 0.194) (Table 3).

On multivariable analysis adjusting for CCI and LOS, preadmission and inpatient opioid use were no longer found to be associated with 90-day readmission, but discharge opioid use was found to increase the risk of 90-day readmission (*p* = 0.002) (Table 3).

### 3.4. Opioid Dose and Readmission

#### 3.4.1. Opioid Dose and 30-Day Readmission

The median home maximum daily dose of opioids among patients with a 30-day readmission was 48.0 MME, compared with 45.0 MME among those without a 30-day readmission. Dosing of preadmission opioids was not found to be associated with 30-day readmissions on either univariable or multivariable analysis, adjusted for CCI and LOS (Table 2). Among admitted patients, the median average daily dose of opioids for those with a 30-day readmission was 7.6 MME, compared with 6.8 MME for those without a 30-day readmission. The daily dose of inpatient opioids was not found to increase the risk of 30-day readmissions (Table 2). Among the 340 patients with inpatient opioid use, 308 (90.6%) had intravenous administration (IV), 155 (45.6%) had oral administration (PO), and 12 (3.5%) had transdermal administration (TD). Mode of opioid administration was not found to differ between those with or without a 30-day readmission.

Among patients with discharge opioid use, the median maximum daily dose was 45.0 MME. The median maximum daily dose for those with a 30-day readmission was 48.0 MME, compared with 45.0 MME for those without a 30-day readmission. Dosing of opioids on discharge was not associated with 30-day readmission on univariable analysis but was associated with 30-day readmission on multivariable analysis, adjusted for CCI and LOS (*p* = 0.037) (Table 2).

#### 3.4.2. Opioid Dose and 90-Day Readmission

Among patients with preadmission opioid use, the median maximum daily dose of opioids was 46.5 MME for those with a 90-day readmission and 45.0 MME for those without a 90-day readmission. Preadmission opioid doses were found to be associated with 90-day readmission on both univariable (*p* = 0.038) and multivariable (*p* = 0.029) analysis (Table 3). Among patients with inpatient opioid use, the median average daily dose of opioid was found to significantly differ between those with a 90-day readmission (8.5 MME) and those without a 90-day readmission (6.3 MME), on univariable analysis (*p* = 0.043) but not on multivariable analysis (*p* = 0.540) (Table 3). Inpatient oral opioid administration was found to be significantly associated with 90-day hospital readmissions on both univariable (*p* = 0.001) and multivariable analysis (*p* = 0.003) (Table 3). The mean maximum daily dose of discharge opioid for those with a 90-day readmission was 72.78 MME, compared with 52.89 MME for those without a 90-day readmission. The median maximum daily dose of discharge opioid was not associated with 90-day readmission on univariable analysis but was significantly associated with 90-day readmission on multivariable analysis (*p* = 0.034) (Table 3).

## 4. Discussion

Hospital readmissions continue to contribute significantly to the high cost of care and diminished QOL among IBD patients. Prior studies have identified risk factors for readmission in this population, such as age, cannabis use, longer LOS during index hospitalization, medical comorbidities, and psychosocial factors [10,11,12,13,14,15,16,17]. Our analysis again identified LOS and increased comorbidities at index admission as associated with readmissions. Opioid use has also drawn scrutiny as a potential risk factor for readmission, but results have been conflicting. To investigate the potential role of opioids, we undertook a comprehensive analysis of opioid use and dosing for a large cohort of medically treated IBD patients, across the spectrum from preadmission, through hospitalization, and at the time of discharge. Our findings do not suggest an increased risk of hospital readmissions when opioids are used as part of inpatient care, as was the case for almost a third of patients observed. However, we did find that patients discharged with an opioid prescription were more likely to have a readmission within 90 days, and that among those receiving opioids at discharge, patients readmitted within both 30 and 90 days tended to be on higher opioid doses.

Though the issue of IBD and opioid use and readmission is well studied, the methodologies and results of prior work have led to differing outcomes. Micic et al. [10] in their study of the Nationwide Readmissions Database (NRD) 2013 observed that opioid dependence (OR: 1.40; 95% CI 1.06–1.86) was associated with an increased risk of 30-day readmission. These findings were also observed by Charilaou et al. [9] in their updated NRB analysis which identified 487,728 IBD cases, of which 6633 [1.4%] had documented opioid use disorder (OUD). Thirty-day readmissions were significantly more common in the OUD group compared to the non-OUD group: 32.6% vs. 19.2%; *p* < 0.001. It is important to note that the smaller proportion of patients with OUD in these studies, as compared to opioid use in our own work, is related to the narrower definition of OUD by an International Classification of Diseases (ICD) coding. In those studies, OUD is a coded medical diagnosis, rather than a direct patient-level observation of opioid use. I.e., use of opioids is far more frequent than a medically defined disorder of use or dependence. Recent population-based studies have suggested an increase in the rates of OUD among IBD patients overall and during hospitalization, though it is unclear if this is a true increase in OUD or rather an increased recognition of the disorder [18,19].

Sheehan et al. [4], in their recent systematic review and meta-analysis, have addressed the issue of opioid use more broadly. Their analysis of opioid use and healthcare utilization in patients with IBD included seven studies involving 4688 patients, which addressed the risk of 30-day readmission. The pooled relative risk (RR) demonstrated no difference in readmission rates in patients receiving opioids compared with those who did not (RR: 1.17; 95% confidence interval: 0.86–1.61). This was true as well in subgroup analysis in those who received opioids prior to admission, during admission, or at discharge. Of the studies included in the meta-analysis, Hazratjee et al. [20] notably found that patients discharged without opioids had a 2.2-fold increased risk of readmissions. Our study also failed to demonstrate an association between opioid use and 30-day readmission, while finding that opioid use at discharge was associated with 90-day readmission. This is not an outcome addressed by the recent meta-analysis, but one that is still a valid cause for concern. While our study was able to access the instructions and prescriptions provided to patients at the time of discharge, we were unable to follow these patients as they transitioned back to outpatient care. Given that prescriptions are often given as a one-month supply, we suspect that readmissions after 30 days and before 90 days may have been due to patients running out of opioids, though this is merely a hypothesis without supporting evidence. This is not an argument for longer courses or increased use of opioids, but rather reinforces the idea that opioids are a clear marker of a sicker patient, and that greater care, both prior to and post-discharge, is necessary to improve outcomes. This is reinforced by our secondary finding that higher opioid doses at discharge impacted both 30- and 90-day outcomes.

Our study is among the few that have examined the association between hospital readmissions and opioid characteristics, including dosing and route of opioid administration. Our findings showed an association between hospital readmissions and the maximum daily dose of opioids at home and on discharge. We also observed an association between the average daily dose of inpatient opioid use and readmissions. These findings emphasize opioid use as a risk factor for increased healthcare utilization while questioning whether it has a dose-dependent effect on future opioid exposure. Dalal et al. explored this idea in a cohort study that examined the association between inpatient opioid exposure and opioid prescriptions written within 12 months post-discharge. Their report showed an association between higher opioid dosing and post-discharge opioid exposure [21].

Overall, the numerous confounding factors of our study may suggest an association between types of opioid use and hospital readmission but cannot be overinterpreted to imply causation. Our study has several limitations that are common to retrospective analyses of data obtained from an electronic health record. Capturing pre-hospitalization opioid exposure and dosing was reliant on the admitting provider’s ability to obtain an accurate medication reconciliation. Though patients prescribed opioids in New York State can all have their prescriptions verified by the state’s prescription monitoring program registry (I-STOP/PMP), accessing this information is not required at the time of admission. Also, though common among IBD patients, the stigma that is still associated with opioid use might cause some patients to underreport their use at home. On the other hand, data on opioid use are more rigorously documented during hospitalization. While the methodology of the study offered the ability to capture average inpatient opioid use, it was still limited in regard to dose calculations from opioid infusions through patient-controlled anesthesia pumps, which may have led to an underestimation of inpatient IV opioid exposure and impacted the study results. As with other studies examining readmission as an outcome, we were limited by our inability to track patients following their discharge. Timeliness of post-discharge follow-up [15] has also been shown to impact readmissions. Our hospitals are not part of a closed healthcare system; therefore, most patients follow up with providers outside of our institution. Further, there are numerous unaffiliated hospitals within close proximity, opening the possibility of undocumented readmissions to outside institutions. Given the inability to access admission data from other organizations, there is no way of estimating how many and what proportions of hospital readmissions may have occurred outside of the Northwell Health system.

While our study was able to assess and adjust for some factors associated with readmission (i.e., LOS, CCI), data such as disease duration, phenotype, and objective disease severity were not calculated during hospitalization and, therefore, could not be incorporated into the analysis. Additionally, the bioinformatics methodology captured all-cause readmission but did not distinguish between readmissions related or unrelated to IBD. Of course, opioid use itself may merely be a marker for more severe disease, itself leading to a higher rate of hospital readmissions. We did, however, take care to only include patients with an IBD flare, limiting inclusion to those with an IBD diagnosis who received inpatient corticosteroid and/or biologic consistent with a disease flare. The exclusion of patients who underwent IBD-related surgery during index hospitalization was intentional and meant to provide a more homogenous group for our analysis.

In conclusion, our analysis of 30- and 90-day hospital readmissions among patients admitted with an IBD exacerbation demonstrated risk factors for readmission, including longer LOS and increased medical comorbidities. We did not demonstrate any association between inpatient opioid use overall and rehospitalization. Our analysis did find an association between 90-day readmission and opioid prescribing at discharge, as well as higher opioid dosing at discharge and both 30- and 90-day readmissions. Our findings provide some reassurance regarding the use of inpatient opioids when our patients need this support most. However, these findings also send a strong message of caution to those discharging patients who still require opioid analgesia. When discharging patients on opioids, we would suggest an objective reassessment as to whether these patients have truly achieved their admission goals of care. Even if admission goals have been achieved, the continued use of opioids at discharge should mandate extra-close follow-up of IBD activity, and the involvement of pain management specialists to aid in opioid de-escalation and to supervise alternative methods for pain management in this population.

## Figures and Tables

**Table 1:** Demographic and clinical characteristics by 30- and 90-day readmission.

Characteristic ^1^	Overall	30-Day Readmission			90-Day Readmission		
	N = 1062 *n* (%)	N = 191 Yes, *n* (%)	N = 871 No, *n* (%)	*p*-Value ^2^	N = 285 Yes, *n* (%)	N = 777 No, *n* (%)	*p*-Value ^2^
Age							
Median (IQR)	43.0 (28.0, 61.0)	47.0 (28.0, 63.0)	43.0 (28.0, 60.0)	0.339	45.0 (29.0, 63.0)	43.0 (27.0, 60.0)	0.118
Gender							
Female	568 (53.5)	101 (52.9)	467 (53.6)	0.853	161 (56.5)	407 (52.4)	0.234
Male	494 (46.5)	90 (47.1)	404 (46.4)		124 (43.5)	370 (47.6)	
Race							
Asian	55 (5.2)	10 (5.2)	45 (5.2)	0.940	13 (4.6)	42 (5.4)	0.492
Black	15 (14.2)	29 (15.2)	122 (14.0)		44 (15.4)	107 (13.8)	
Other/Multiracial	143 (13.5)	23 (12.0)	120 (13.8)		32 (11.2)	111 (14.3)	
White	679 (63.9)	124 (64.9)	555 (63.7)		184 (64.6)	495 (63.7)	
Unknown	34 (3.2)	5 (2.6)	29 (3.3)		12 (4.2)	22 (2.8)	
CCI							
Median (IQR)	1.0 (0.0, 3.0)	1.0 (0.0, 5.0)	1.0 (0.0, 3.0)	0.010	1.0 (0.0, 4.0)	1.0 (0.0, 3.0)	<0.001
IBD Type							
Crohn’s Disease	501 (47.2)	98 (51.3)	403 (46.3)	0.418	148 (51.9)	353 (45.4)	0.116
Ulcerative Colitis	537 (50.6)	90 (47.1)	447 (51.3)		133 (46.7)	404 (52.0)	
Other	24 (2.3)	3 (1.6)	21 (2.4)		4 (1.4)	20 (2.6)	
LOS (Days)							
Median (IQR)	4.4 (2.8, 7.3)	5.9 (3.7, 11.3)	4.1 (2.6, 6.8)	<0.001	5.7 (3.5, 10.1)	4.0 (2.6, 6.7)	<0.001
Anxiety							
Yes	110 (10.4)	19 (9.9)	91 (10.4)	0.837	33 (11.6)	77 (9.9)	0.429
No	952 (89.6)	172 (90.1)	780 (89.6)		252 (88.4)	700 (90.1)	
Depression							
Yes	68 (6.4)	10 (5.2)	58 (6.7)	0.467	20 (7.0)	48 (6.2)	0.620
No	994 (93.6)	181 (94.8)	813 (93.3)		265 (93.0)	729 (93.8)	
Alcohol Use							
Current	185 (17.4)	26 (13.6)	159 (18.3)	0.338	42 (14.7)	143 (18.4)	0.563
Former	25 (2.4)	6 (3.1)	19 (2.2)		7 (2.5)	18 (2.3)	
None	426 (40.1)	76 (39.8)	350 (40.2)		120 (42.1)	306 (39.4)	
Unknown	426 (40.1)	83 (43.5)	343 (39.4)		116 (40.7)	310 (39.9)	
Tobacco Use							
Current	103 (9.7)	17 (8.9)	86 (9.9)	0.311	33 (11.6)	70 (9.0)	0.178
Former	179 (16.9)	41 (21.5)	138 (15.8)		56 (19.6)	123 (15.8)	
None	643 (60.5)	109 (57.1)	534 (61.3)		165 (57.9)	478 (61.5)	
Unknown	137 (12.9)	24 (12.6)	113 (13.0)		31 (10.9)	106 (13.6)	

^1^ IQR = interquartile range, CCI = Charlson Comorbidity Index, IBD = inflammatory bowel disease, LOS = length of stay. ^2^ Pearson’s chi-square test, Fisher’s exact test, Wilcoxon rank sum test.

**Table 2:** Association between opioid use and characteristics with 30-day readmissions.

Characteristic ^1^	Overall	30-Day Readmission				
		Unadjusted			Adjusted	
	*n* (%)	Yes, *n* (%)	No, *n* (%)	*p*-Value ^2^	OR (95% CI) ^3^	*p*-Value ^4^
Home Opiate Use (*N* = 1062)						
Yes	96 (9.0)	25 (13.1)	71 (8.2)	0.031	1.57 (0.96, 2.58)	0.073
No	966 (91.0)	166 (86.9)	800 (91.8)		(ref)	
Home Maximum Daily Dose (MME) (*N* = 96)						
Median (IQR)	45.0 (30.0, 60.0)	48.0 (30.0, 96.0)	45.0 (30.0, 60.0)	0.069	1.01 (1.00, 1.02)	0.054
Inpatient Opiate Use (*N* = 1062)						
Yes	340 (32.0)	70 (36.6)	270 (31.0)	0.130	1.17 (0.84, 1.65)	0.351
No	722 (68.0)	121 (63.4)	601 (69.0)		(ref)	
Inpatient Average Daily Dose (MME) (*N* = 340)						
Median (IQR)	7.0 (2.8, 18.9)	7.6 (3.9, 27.3)	6.8 (2.6, 17.3)	0.245	1.00 (1.00, 1.01)	0.484
Inpatient IV Administration Mode (*N* = 340)						
Yes	308 (90.6)	63 (90.0)	245 (90.7)	0.850	1.15 (0.44, 3.04)	0.775
No	32 (9.4)	7 (10.0)	25 (9.3)		(ref)	
Inpatient PO Administration Mode (*N* = 340)						
Yes	155 (45.6)	37 (52.9)	118 (43.7)	0.171	1.25 (0.66, 2.33)	0.492
No	185 (54.4)	33 (47.1)	152 (56.3)		(ref)	
Inpatient TD Administration Mode (*N* = 340)						
Yes	12 (3.5)	3 (4.3)	9 (3.3)	0.717	0.73 (0.14, 3.85)	0.712
No	328 (96.5)	67 (95.7)	261 (96.7)		(ref)	
Discharge Opiate Use (*N* = 1062)						
Yes	132 (12.4)	34 (17.8)	98 (11.3)	0.013	1.48 (0.95, 2.29)	0.081
No	930 (87.6)	157 (82.2)	773 (88.7)		(ref)	
Discharge Maximum Daily Dose (MME) (*N* = 132)						
Median (IQR)	45.0 (30.0, 64.0)	48.0 (30.5, 90.0)	45.0 (30.0, 63.0)	0.105	1.01 (1.00, 1.02)	0.037

^1^ IQR = interquartile range, IV = intravenous, PO = by mouth, TD = transdermal, MME = morphine milligram equivalents. ^2^ Pearson’s chi-square test, Fisher’s exact test, Wilcoxon rank sum test. ^3^ OR = odds ratio, CI = confidence interval, ref = reference group. ^4^ Multivariable logistic regression, adjusted for the Charlson Comorbidity Index and length of stay.

**Table 3:** Association between opioid use and characteristics with 90-day readmissions.

Characteristic ^1^	Overall	90-Day Readmission				
		Unadjusted			Adjusted	
	*n* (%)	Yes, *n* (%)	No, *n* (%)	*p*-Value ^2^	OR (95% CI) ^3^	*p*-Value ^4^
Home Opiate Use (*N* = 1062)						
Yes	96 (9.0)	34 (11.9)	62 (8.0)	0.047	1.43 (0.92, 2.26)	0.114
No	966 (91.0)	251 (88.1)	715 (92.0)		(ref)	
Home Maximum Daily Dose (*N* = 96)						
Median (IQR)	45.0 (30.0, 60.0)	46.5 (30.0, 94.5)	45.0 (30.0, 60.0)	0.038	1.01 (1.00, 1.03)	0.029
Inpatient Opiate Use (*N* = 1062)						
Yes	340 (32.0)	100 (35.1)	240 (30.9)	0.194	1.13 (0.84, 1.51)	0.442
No	722 (68.0)	185 (64.9)	537 (69.1)		(ref)	
Inpatient Average Daily Dose (*N* = 340)						
Median (IQR)	7.0 (2.8, 18.9)	8.5 (4.2, 26.2)	6.3 (2.5, 17.0)	0.043	1.00 (1.00, 1.01)	0.540
Inpatient IV Administration Mode (*N* = 340)						
Yes	308 (90.6)	91 (91.0)	217 (90.4)	0.867	1.97 (0.80, 4.85)	0.138
No	32 (9.4)	9 (9.0)	23 (9.6)		(ref)	
Inpatient PO Administration Mode (*N* = 340)						
Yes	155 (45.6)	59 (59.0)	96 (40.0)	0.001	2.32 (1.31, 4.05)	0.003
No	185 (54.4)	41 (41.0)	144 (60.0)		(ref)	
Inpatient TD Administration Mode (*N* = 340)						
Yes	12 (3.5)	5 (5.0)	7 (2.9)	0.346	1.26 (0.28, 5.61)	0.757
No	328 (96.5)	95 (95.0)	233 (97.1)		(ref)	
Discharge Opiate Use (*N* = 1062)						
Yes	132 (12.4)	54 (18.9)	78 (10.0)	<0.001	1.86 (1.27, 2.75)	0.002
No	930 (87.6)	231 (81.1)	699 (90.0)		(ref)	
Discharge Maximum Daily Dose (*N* = 132)						
Median (IQR)	45.0 (30.0, 64.0)	45.0 (30.0, 90.0)	45.0 (30.0, 60.0)	0.112	1.01 (1.00, 1.02)	0.034

^1^ IQR = interquartile range, IV = intravenous, PO = by mouth, TD = transdermal. ^2^ Pearson’s chi-square test, Fisher’s exact test, Wilcoxon rank sum test. ^3^ OR = odds ratio, CI = confidence interval, ref = reference group. ^4^ Multivariable logistic regression, adjusted for the Charlson Comorbidity Index and length of stay.

## Data Availability

The data underlying this article cannot be shared publicly for the privacy of individuals who participated in the study. The data will be shared upon reasonable request to the corresponding author.

## Abbreviations

CCI	Charlson Comorbidity Index
CD	Crohn’s Disease
CI	Confidence Interval
ER	Emergency Room
IBD	Inflammatory Bowel Disease
ICD-10	International Classification of Diseases, 10th Revision
IQR	Interquartile Range
IRB	Institutional Review Board
IV	Intravenous
LOS	Length of Stay
MME	Morphine Milligram Equivalent
OR	Odds Ratio
OUD	Opioid Use Disorder
PMP	Prescription Monitoring Program
PO	By Mouth (Per Os)
QOL	Quality of Life
RR	Relative Risk
TD	Transdermal
